# An overview of reviews of Angiotensin II in distributive shock

**DOI:** 10.1186/s12871-026-03797-w

**Published:** 2026-04-06

**Authors:** Mathieu Desfosses, Jo Røislien

**Affiliations:** 1 Faculty of Health Sciences, University of Stavanger, Stavanger, Norway; 2 Kamloops, British Columbia V1S0B1 Canada; 3 Department of Research, The Norwegian Air Ambulance Foundation, Oslo, Norway

**Keywords:** Angiotensin II, Distributive shock, Septic shock, Vasoplegia, Critical care

## Abstract

**Backgroud:**

High-dose catecholamine use in distributive shock is associated with high mortality. Angiotensin II (ATII) has demonstrated benefits in distributive shock patients, yet limited and inconsistent clinical data precludes its publication in treatment guidelines. This overview of reviews aims to critically assess and summarize systematic reviews on ATII benefits in distributive shock, identify knowledge gaps, and provide recommendations for future research.

**Method:**

A systematic literature search for systematic reviews on the topic from 2020 was conducted, including PubMed, Embase, CINAHL, Epistemonikos, Scopus, PROSPERO, and Cochrane, and gray literature. Reviews were assessed by the Assessment of Multiple Systematic Reviews 2 (AMSTAR 2) checklist. Certainty of evidence was calculated using the Grading of Recommendations Assessment, Development and Evaluation (GRADE) tool. Both qualitative and quantitative data were summarized.

**Results:**

The literature search yielded 239 results, resulting in 6 systematic reviews being included. The methodological quality was mostly low. ATII administration was associated with a decrease in background vasopressor requirements. Occurrence of serious adverse reactions were not found to be statistically significant. Mortality benefits was found in certain patient sub-groups but cannot be generalized. A consistent finding was that ATII improved MAP > 10 mmHg from baseline.

**Conclusion:**

ATII appears to be a safe and effective vasopressor in distributive shock management. The physiological and logistical benefits should be considered early in distributive shock management. This overview of reviews has identified certain gaps in the definition of catecholamine-resistant shock, ATII initiation thresholds, biomarker guided treatment, and occurrence of hemodynamic relapse which may guide future aspiring clinical trials.

**Supplementary Information:**

The online version contains supplementary material available at 10.1186/s12871-026-03797-w.

## Introduction

Distributive shock, or vasoplegia, is one of the leading causes of intensive care unit (ICU) admission with a mortality rate of 30–50% [1]. Arterial vasodilation and hypotension from distributive shock impairs oxygen delivery, leading to cellular injury and organ failure. Fluid administration and vasopressors are cornerstones for re-establishing vascular tone and tissue perfusion [1]. Catecholamine refractory shock (CRS) refers to a severe subset of distributive shock patients, characterized by hypoperfusion despite adequate fluid resuscitation and vasopressor treatment [2]. As a result, patients necessitate higher doses of vasopressors and additional interventions. This poses concerns as escalating norepinephrine over 0.5mcg/kg/min has been associated with mortality rates to 60–90% [1].

Angiotensin II (ATII) is an endogenous key effector of the renin–angiotensin–aldosterone system, and has been suggested as an adjunct in refractory shock management [3]. The ATHOS-3 trial [4] demonstrated ATII's effectiveness in increasing mean arterial pressure (MAP) in patients resistant to high-dose vasopressors, and resulted in FDA and EMA approval for septic shock treatment [5]. Yet, the current literature offers mixed conclusions about its efficacy. Systematic reviews have evaluated ATII for distributive shock; however, these reviews vary in methodological quality, inclusion criteria, and outcome reporting, leading to heterogeneity in their conclusions. Consequently, the overall state of evidence remains difficult to interpret. To date, no comprehensive synthesis of these systematic reviews exists. This overview of reviews therefore assesses the methodological quality of existing systematic reviews and summarizes ATII’s impact on vasopressor requirements, adverse events, mortality, administration practices, and MAP improvement.

## Methods

This overview of reviews was conducted following the Preferred Reporting Items for Overviews of Reviews (PRIOR) statement [6]. (Appendix A). Predetermined protocol was registered in PROSPERO (CRD42024590787), from which we report no deviation.

### Eligibility criteria

This overview includes systematic reviews addressing the clinical effects of ATII in distributive shock. Other types of shock were not included due to etiopathogenic and management discrepancies. As distributive shock exists on a spectrum and may involve other organs leading to a mixed shock state, a pragmatic approach was adopted requiring review characteristics and primary studies to meet the pre-determined physiological criteria to ensure the shock state was distributive in nature. Comparators of ATII use included placebo once standards-of-care (SOC) thresholds had been met or SOC alone. We deemed this physiologically comparable as ATII was administered in comparison to a predetermined threshold of SOC. This rationale was subsequently supported following correspondence with the authors of the included systematic reviews. Systematic reviews and primary studies could thus be vetted based on the research question. Review outcomes required reporting at least one of the following: changes in MAP following ATII administration, effects on background vasopressor requirements or catecholamine dose reduction, mortality, occurrence of serious adverse events associated with ATII, or reported ATII dosing practices. Eligibility criteria are shown in Table 1.

**Table 1:** Summary of the inclusion and exclusion criteria of this overview

Study Criteria	Inclusion Criteria	Exclusion Criteria
Study design	Systematic review (± meta-analysis) *We defined systematic reviews as any review that uses reproducible methods to systematically search, critically appraise, and synthesize studies on a specific issue*	Non-reviews, protocols only, narrative reviews, abstract only, editorials, opinion articles with inadequate or no proper data on the topic, reviews labelled as ongoing, and other types of reviews which did not use a systematic search strategy
Population	Distributive shock or vasoplegic shock defined as difficulty in maintaining mean arterial pressure (MAP) above 65 mmHg despite standards of care (SOC) with preserved cardiac index (CI) of at least 2.2 L/min/m^2^	Shock not distributive in nature. Mixed shock states
Intervention	Administration of Angiotensin II (Giapreza) once SOC thresholds have been met	Non-Angiotensin II administration
Comparator (Control)	Standards of care defined as intravenous volume resuscitation of 25 ml/kg and vasopressor requirements and/or Placebo once SOC goals have been met	Patients not receiving current standards of care prior to ATII initiation
Timing	Reviews published after 2020 comprised of primary studies published after 2014	Reviews reflective of outdated use and formulation of ATII, primarily comprised of studies prior to 2014
Outcome	Effects on background vasopressor requirements, occurrence of serious adverse reactions, mortality, ATII dose, and effects on MAP	Any outcome unrelated to hemodynamic or safety profile

### Search strategy

A comprehensive search was conducted across PubMed, Embase, CINAHL, Epistemonikos, Scopus, PROSPERO, and Cochrane, supplemented by manual gray literature screening. The search strategy was limited to English language reviews from 2020 onwards, reflective of modern use of ATII following its resurgence since the 1990’s. The search included the following terms in combination: Angiotensin II, or ATII, or Angiotensin 2, and Distributive Shock, or Vasoplegia, or Shock, or Hypotension. Systematic review was sourced out by term search or filter application. A final search was completed on March 15, 2025. Supplementary data on the search and review selection can be found in Appendix B.

### Study selection

Two reviewers (MD, HA) independently screened titles and abstracts of selected studies. A university librarian performed a third independent search. Rayyan AI [7] was used to manage the studies. Duplicates were automatically detected and deleted. A full-text review to assess eligibility was then performed by the lead reviewer (MD). Acceptable primary studies within the reviews included 2014 publications onwards. Where outlying primary studies were identified in selected reviews a pre-determined decision rule was established to assess the effect of the study within the review for potential internal validity impacts; outcomes heavily weighted on such studies were excluded for this overview. Two reviewers (MD, HA) subsequently retrieved primary studies from the reviews to ensure adherence to the criteria. Any disagreements on review selection were resolved by discussion and mutual consensus, with arbitration by research supervisor (JR), if required.

### Data collection

Data extraction was performed independently by two reviewers (MD, HA) from the included reviews. A form was designed in Microsoft Excel [8] to collect pertinent data. Quantitative outcome data were also captured. Primary studies within the reviews were also collected for overlap mapping. In addition, we make known the outcomes of interest, conclusions, and recommendations for each review. Overlap was presented visually using the Graphical Representation of Overlap of OVErviews (GROOVE2.0) tool [9] (Appendix C). Discrepant data were assessed on a case-by-case basis and the root cause identified. In instances where data were missing, the primary investigator contacted the original review authors. Where additional data could not be provided, extraction of only available data was included.

### Data items

Key outcomes include ATII’s impact on background vasopressor requirements and occurrence of serious adverse reactions. We also include occurrence of the most reported adverse reactions within systematic review, defined as an occurrence or clinical deterioration associated with the use of ATII, from randomized controlled trials (RCTs) and observational studies. Additionally, ATII dosing, mortality, and hemodynamic effects on MAP such as vasopressor trends and hemodynamic profiles were collected. These outcomes were extracted and reported according to the definitions used within the included systematic reviews. We acknowledge that outcome measures varied across reviews; for example, MAP response was defined either as achieving a target ≥ 65 mmHg or as an increase from baseline.

### Methodological quality and risk of bias

The methodological quality of the reviews was assessed using the Assessment of Multiple Systematic Reviews-2 (AMSTAR2) [10] checklist (Appendix D). Authors of the included reviews were contacted to provide complementary data when required.

Risk of bias (ROB) of primary studies was collected from systematic reviews when provided. Reproduction of ROB assessments was done with permission from lead authors. Missing ROB assessments were sourced by correspondence with lead authors. When authors did not respond, ROB assessment was performed anew by two reviewers (MD, HA) independently. The Risk of Bias In Non-randomized Studies of Interventions ROBINS-I tool [11] was used for observational studies and Risk of Bias 2 tool [12] was used for RCTs (Appendix E).

### Synthesis methods

Due to non-standardized reporting of quantitative summary measures (means, medians, SDs, risk ratios) in the systematic reviews, summarizing the summary measures across the included papers using statistical methods was not possible. Therefore, no meta-analysis was conducted. As there was also considerable overlap in primary studies, findings are reported narratively. Primary study overlap among the included reviews was quantified by the primary reviewer (MD) and verified by a second reviewer (HA) using the corrected covered area (CCA) index [9, 13]. Overlap was assessed at the outcome level across included reviews. Predetermined thresholds from the GROOVE 2.0 tool were used to interpret the degree of overlap. In addition to the CCA calculation, visualization of overlap using GROOVE 2.0 [9] was performed to provide a graphical representation of how primary studies contributed across reviews and outcomes, allowing clearer interpretation of the distribution and potential influence of overlapping evidence. When consistent, we present outcomes from reviews with the least amount of overlap, with the greater number of primary studies, and with the most recent studies. For discordant data or variability, we sought to identify factors that potentially influenced the outcomes of interest. The outcomes of the reviews are then presented narratively.

### Certainty of evidence

Outcomes were evaluated using the Grading of Recommendations, Assessment, Development, and Evaluations (GRADE) [14] framework. The certainty of evidence for each outcome was assessed by two review authors (MD, HA). GRADE results range from very low to high based on: judgment of the risk of bias, inconsistency, indirectness, imprecision, and publication bias. As the tool is not yet adapted for overviews, pre-determined rules for downgrading certainty of evidence were established based on criteria from the GRADE Handbook [14].

## Results

### Study selection

**Fig. 1:**
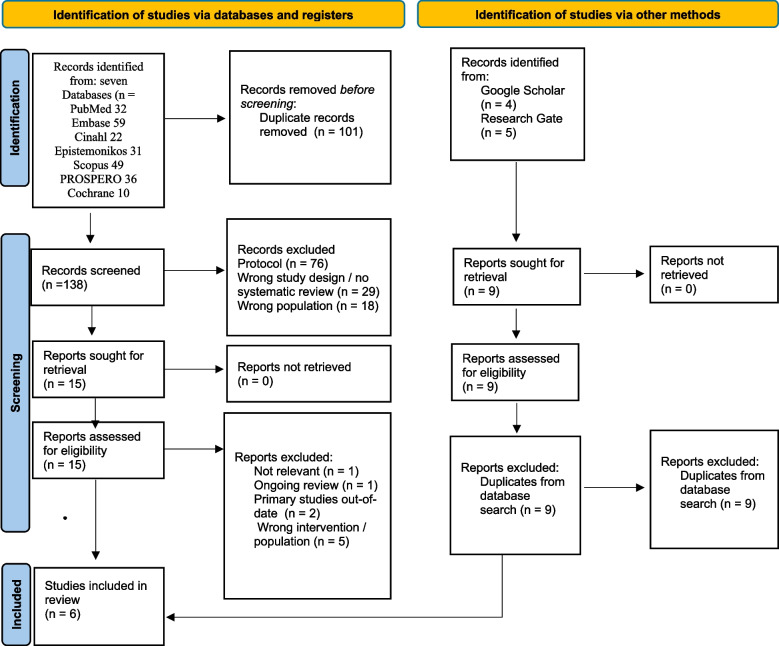
Preferred Reporting Items for Systematic Reviews and Meta-Analyses (PRISMA) [15] flow diagram of systematic reviews identified, excluded, and included

A total of 239 records were identified in the initial search from databases and an additional 9 results from gray literature. Of these, 110 duplicates were removed. The remaining 138 studies were screened with title and abstract based on inclusion and exclusion criteria, of which 123 were excluded for either not meeting the protocol or wrong study design or patient population. The remaining 15 reviews were retrieved and screened in full text. Of these 9 reviews were excluded: one was not relevant to the inclusion criteria, one was ongoing, two were comprised primarily of out-of-date primary studies, and five represented the wrong patient populations. From this screening process, a total of six reviews were included (Fig. 1). Included reviews are summarized in Table 2. Excluded and included review profiles are presented in Appendix B.

**Table 2:** Characteristics of systematic reviews

Title	Author	Year	Date last assessed as up to date	Number of included studies	Pathology reviewed
The Use of Angiotensin II for the Treatment of Post-cardiopulmonary Bypass Vasoplegia	Papazisi et al. [16]	2020	October 21 st, 2020	9	Post-CPB Vasoplegia *
How Effective is Angiotensin II in Decreasing Mortality of Vasodilatory Shock? A Systematic Review	Semedi et al. [17]	2023	January 5th, 2023	4	Sepsis and post-CPB vasoplegia
Angiotensin II in the Treatment of Distributive Shock: A Systematic Review and Meta-Analysis	Xourgia et al. [18]	2024	April 24th, 2024	10	Sepsis and post-CPB vasoplegia
Clinical Outcomes of Angiotensin II Therapy in Vasoplegic Shock: A Systematic Review and Meta-Analysis	Alamami et al. [19]	2024	August 29th, 2024	8	Sepsis and post-CPB vasoplegia
Angiotensin-II and Thromboembolic Events: A Systematic Review	Caragata et al. [20]	2024	October 16th, 2024	7	Sepsis and post- CPB vasoplegia
The efficacy and safety of angiotensin II for treatment of vasoplegia in critically ill patients: a systematic review	Kotani et al. [21]	2024	December 18th, 2024	59	Sepsis and post-CPB vasoplegia *

*Abbreviations*: *CPB* Cardio-Pulmonary Bypass surgery

^*^Identified studies included in the reviews which did not meet the pre-determined criteria

### Study characteristics

The most common etiologies of distributive shock in the included systematic reviews were septic shock and post-CPB vasoplegia. The 3-h post administration timeframe was a consistent finding across all reviews in the evaluation of the effects of ATII on background vasopressors and MAP. Papazisi et al. [16] included three studies that were outdated, whereas Kotani et al. [21] included eight. Specifically*,* these excluded trials were published between 1966 and 2001, which does not reflect the modern use and available formulation of ATII [22]. These studies were used to reference the main body of the reviews rather than data synthesis. Two of the thirty-four primary studies could not be retrieved due to lack of access (Appendix F). Fifteen studies which did not meet the predefine pathophysiological criteria were included in the review by Kotani et al. [21]. During data extraction these were isolated as not to impact the outcomes for this overview.

### Primary study overlap

**Fig. 2:**
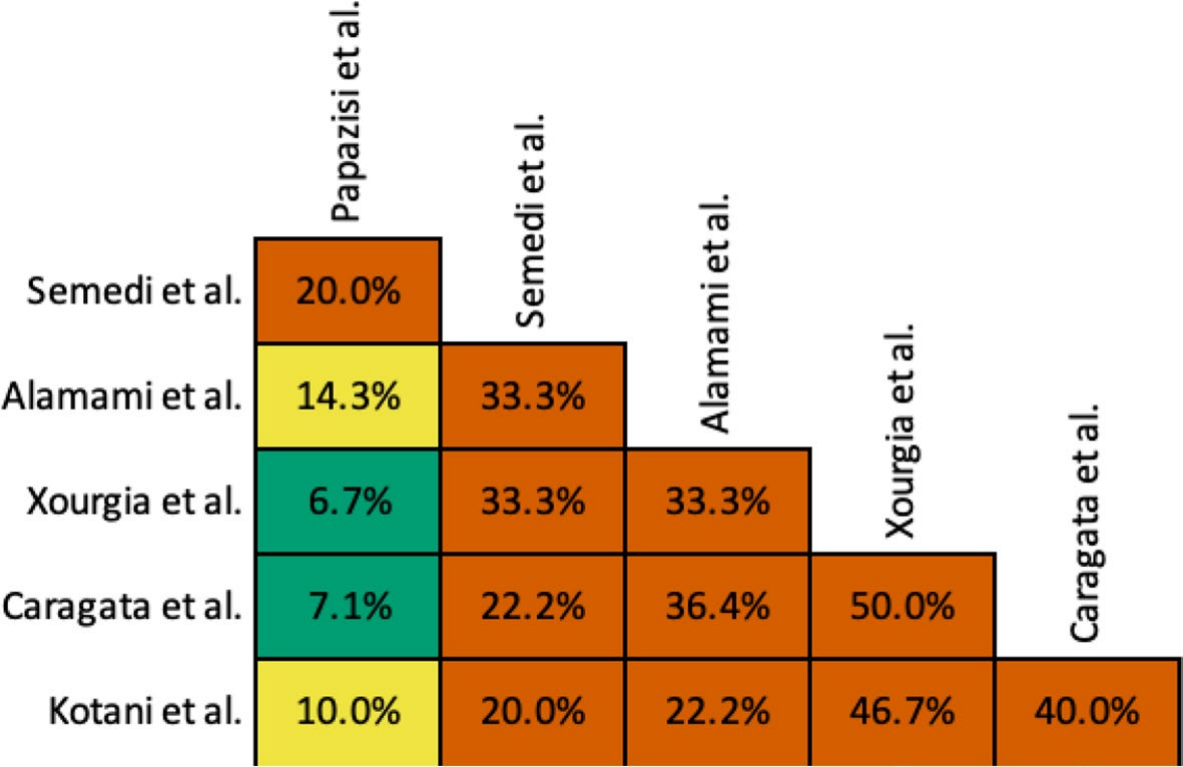
Corrected Covered Area (CCA) of primary study overlap amongst included systematic reviews. White boxes indicate slight overlap (< 5%), green boxes indicate moderate overlap (5%−10%), yellow boxes indicate high overlap (10%−15%), and orange boxes indicate very high overlap (> 15%)

**Fig. 3:**
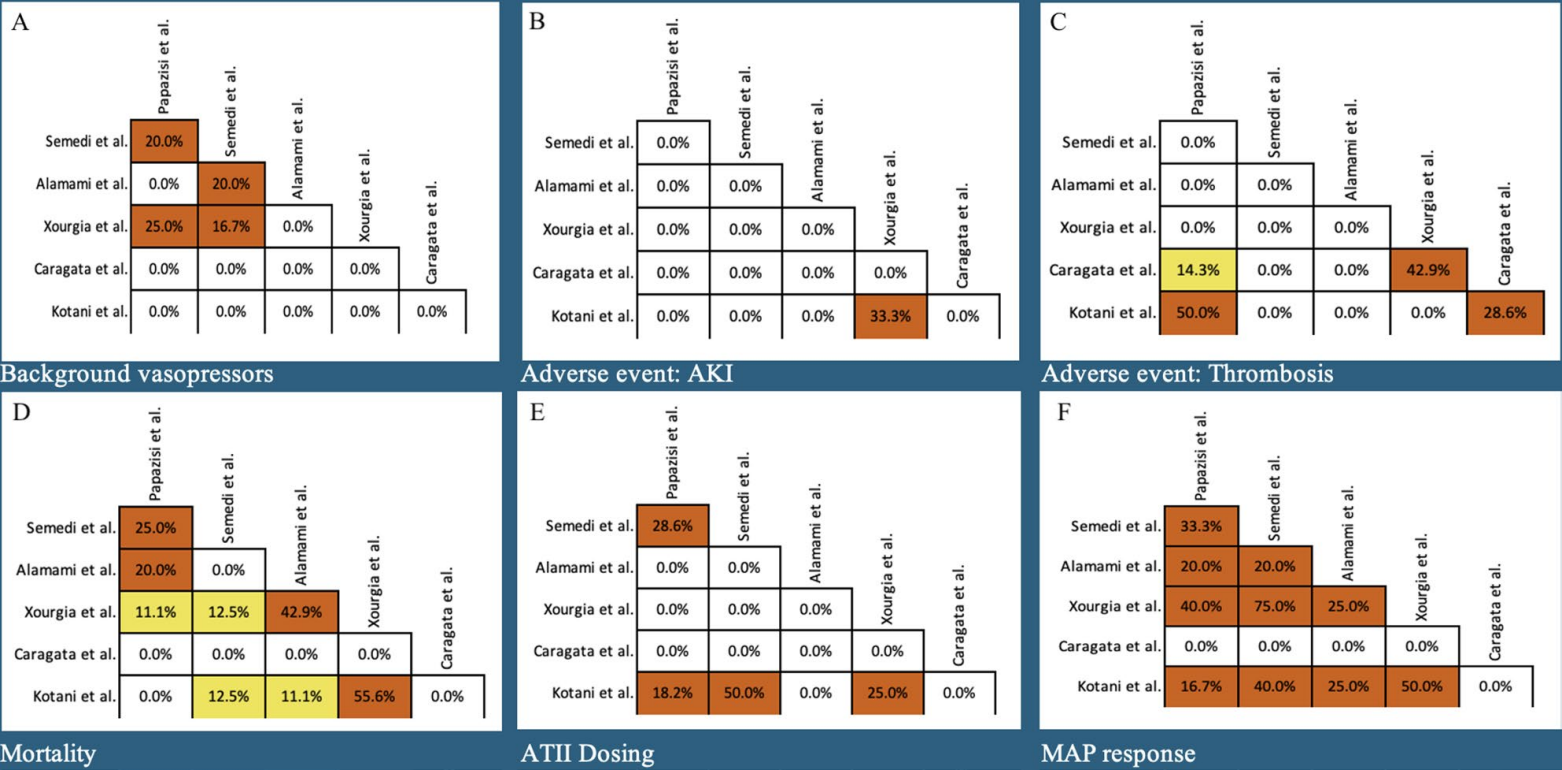
Primary study overlap amongst systematic reviews for outcomes of interest; background vasopressor profiles (box **A**), risk of acute kidney injury (box **B**), risk of thrombosis (box **C**), mortality (box **D**), ATII dosing (box **E**), MAP response (box **F**). White boxes indicate slight overlap (<5%), green boxes indicate moderate overlap (5%-10%), yellow boxes indicate high overlap (10%-15%), and orange boxes indicate very high overlap (>15%)

There was a considerable overlap of primary studies amongst the included reviews (Fig. 2), with a CCA of 20% (Appendix C). Despite high overlap, data mapping found variable overlap depending on the outcome assessed (Fig. 3).

### Quality of evidence

One of the six reviews had moderate quality, four had low quality, and one had critically low quality (Table 3). Correspondence was attempted with six authors to provide more data, only two of which responded. None of the six studies contained all of the AMSTAR2 items (Table 3).

**Table 3:**
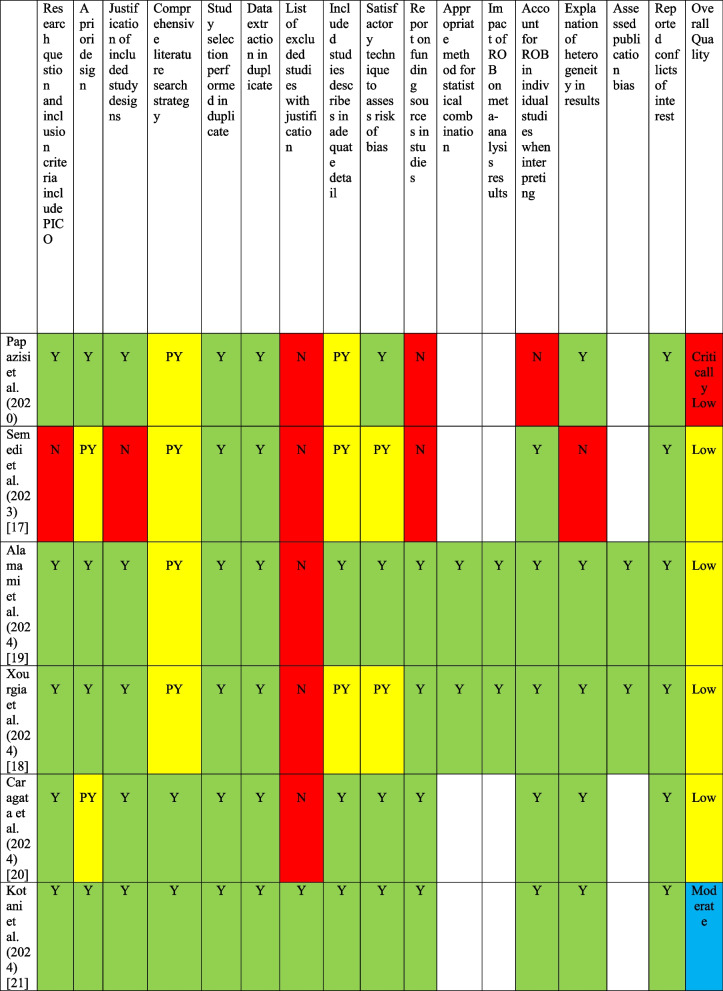
Quality assessment [16–21] based on the Assessment of Multiple Systematic Reviews 2 (AMSTAR-2) tool [10]

Empty cells identify that no meta-analysis is conducted by the authors

*Abbreviations*: *N* No, *Y* Yes, *PY* Partial Yes

### Risk of bias of primary studies

Five of the six systematic reviews provided a ROB assessment of their primary studies. In the one review that did not include a ROB of primary studies, the authors did not respond to our attempts to contact them for data follow-up. Missing results were assessed anew. ROB obtained and assessed are presented in Appendix E.

### Main findings

#### Background vasopressor profiles

Four of the six reviews examined the impact of ATII on background vasopressor requirements, and early initiation was associated with superior reductions in both dose and quantity of vasopressor requirements [16–19]. One meta-analysis reported significantly lower vasopressor requirements at the 3-h mark when ATII was initiated at low norepinephrine equivalent dose (NED, < 200 ng/kg/min) compared to high NED requirements (> 200 ng/kg/min, *p =* 0.01), corresponding to background vasopressor reduction between −130 to −160 ng/kg/min (*p < *0.001) [19]. Similarly, patients receiving ≤ 3 vasopressors at the time of ATII initiation showed greater NED reduction compared to those receiving > 3 (*p =* 0.04). One review quantified change in NED requirements as 580 ± 380 ng/kg/min prior to ATII, followed by a decrease of −200 ng/kg/min at the 3-h mark compared to + 40 ng/kg/min compared to placebo (*p < *0.001) [17]. Patients in early stages of shock were more sensitive to ATII administration, resulting in reduction or discontinuation of NED requirements [16]. ATII was typically used as a third or fourth-line agent.

#### Serious adverse reactions

One systematic review reported similar thrombotic rates between ATII and control groups, attributing events partly to ICU factors, declaring that existing evidence is insufficient to conclude [20]. There were no significant association with ATII and atrial fibrillation when compared to controls [19]. Acute kidney injury (AKI) outcomes were reported in 2 reviews, [18, 21] identifying no higher occurrence of new or worsening AKI or requirement for renal replacement therapy (RRT) compared to SOC therapy [21]. There was no overlap for atrial fibrillation as it was only included by one meta-analysis.

#### Mortality

Mortality outcomes varied, and direct mortality benefits remain unclear [21]. Two meta-analyses reported similar in-hospital mortality between ATII and controls, [18, 19] of which one attributed results to short follow-up periods, [19] while the other claimed no all-cause mortality differences despite the timing of secondary vasopressor administration and NED reduction being closely related to patient outcomes [18]. One review found variation amongst the included studies, identifying mortality benefits from renin–angiotensin–aldosterone system (RAAS) support on several organs and improved oxygenation. ATII was found to have a lower mortality rate (51.1% compared to 69.9% in those receiving placebo, *p =* 0.01) [17]. One review noted that mortality rates in the primary studies were not statistically significant when compared to SOC treatments, yet found sub-populations in which 28-day mortality was influenced. AKI patients receiving RRT who received ATII had improved survival when compared to placebo, and patients sensitive to ATII showed higher survival rates at day 28 compared to non-responders (67% vs. 41%) [16]. Three reviews identified biomarker association with mortality [16–18]. High ATI/ATII ratios were found to be a as significant predictor of mortality (HR 0.54, *p =* 0.011), and elevated renin concentrations were identified as an independent risk factor for mortality (*p =* 0.0013) [16]. Elevated renin was associated with higher mortality (HR 2.15; 95% CI 1.35–3.42); however, in such patients the use of ATII was associated with lower mortality compared to SOC or placebo (HR 0.62; 95% CI, 0.39–0.98) [17]. Influential factors included hemodynamic responsiveness, which was associated with better outcomes (HR 0.50, 95% CI 0.35–0.71, *p < *0.001), whereas higher vasopressor requirements at ATII initiation was associated with worse outcomes (HR 1.61, 95% CI 1.03–2.51, *p =* 0.037) [17].

#### ATII dosing

Initial ATII dose recommendations ranged from 5 to 20 ng/kg/min, with a maximum dose ranging from 40 to 200 ng/kg/min [16–18, 21]. Interestingly, patients with endogenous ATII deficiency, identified as elevated ATI/ATII ratio, responded to lower doses of ATII (< 5 ng/kg/min) correlating to reduced vasopressor needs [16]. One review reported mean administration time as 37 h (range 8 h to 10 days) [21]. However, none of the reviews provided dose to effect trend data, or dosages at which MAP goals were obtained.

#### Effects on mean arterial pressure

Effects of ATII on MAP was reported in five reviews, all indicating that ATII administration improved MAP in patients with distributive shock [16–19, 21]. One review claimed 90% of patients reached the target MAP of 75 mmHg compared to 0% in the control groups; ACE-deficient patients in early stages of shock showing greater sensitivity to early administration of lower doses of ATII (89.9% vs. 51.2% response) [16]. These findings were supported by a meta-analysis claiming 61.2% of patients unresponsive to first- and second-line vasopressors achieved adequate perfusion with the addition of ATII [18]. A target MAP of 75 mmHg was a consistent goal in the studies, yet not always attained. However, a MAP increase of at least 10 mmHg from baseline across every study was noted (*p < *0.001) [17, 19, 21]. Lower lactate levels were associated with statistically significant better hemodynamic responses (*p < *0.001) [16–18]. One review specified MAP improvements in patients with RAAS suppression from angiotensin-converting enzyme inhibitor (ACEi) exposure. However, MAP response was supressed in patients taking angiotensin receptor blockers (ARB) [21]. Similarly, another review and meta-analysis claimed ARB exposure as a negative predictor of MAP obtainment (OR = 0.24; 95% CI: 0.07 to 0.79, *p =* 0.02), suggesting higher doses of ATII are required to reach target MAP in patients with RAAS inhibition due to ARB use [18].

#### Reporting biases

The effect of ATII on MAP was consistently demonstrated across reviews and primary studies [23–25]. However, heterogeneity existed in intervention timing and illness severity reporting. Primary studies were generally low quality, with inconsistent mortality follow-up. Key data were often missing, including the extent of prolonged hypotension, duration of vasopressor use before ATII initiation, and underlying disease states.

#### Certainty of evidence

Assessing the certainty of evidence of the outcomes [14], we found that ATII’s effect on reducing the requirements for background vasopressors and improvement in MAP had high certainty. The relationship between ATII and the adverse effects of thrombosis and atrial fibrillation both had moderate certainty, while the certainty of the effect of ATII on mortality was low (Table 4).

**Table 4:** Certainty of evidence

Outcome	Study design	Risk of bias	Inconsistency	Indirectness	Imprecision	Publication bias	Overall
Background Vasopressor Dose	3 Systematic Reviews	Not serious	Not serious	Not serious	Not serious	Not serious	⨁⨁⨁⨁
Deep Vein Thrombosis	4 Systematic Reviews	Not serious	Not serious	Not serious	Serious*	Not serious	⨁⨁⨁◯
Atrial Fibrillation	1 Systematic review and 1 Meta-analysis	Not serious	Not serious	Not serious	Serious*	Not serious**	⨁⨁⨁◯
Acute Kidney Injury	3 Systematic review and 1 Meta-analysis	Not serious	Not serious	Not serious	Serious*	Not serious**	⨁⨁⨁◯
Mortality	5 Systematic Reviews and 2 Meta-analysis	Not serious	Serious	Not serious	Serious*	Not serious	⨁⨁◯◯
Angiotensin II dose	4 Systematic Reviews and 1 Meta-analysis	Not serious	Not serious	Not serious	Not serious*	Not serious	⨁⨁⨁⨁
Effects on MAP	5 Systematic Reviews including 2 Meta-analysis	Not serious	Not serious	Not serious	Not serious*	Not serious	⨁⨁⨁⨁

^*^Secondary outcomes for this overview

**Paucity in systematic reviews rendering accurate judgement difficult

⨁⨁⨁⨁ = High, ⨁⨁⨁◯ = Moderate, ⨁⨁◯◯ = Low, ⨁◯◯◯ = Very low

## Discussions

This overview of systematic reviews identified 6 reviews evaluating the clinical effects of ATII in distributive shock, occurring in the settings of sepsis and post-CPB vasoplegia. The outcomes of interest with the most evidence supports that ATII administration improves MAP by at least 10 mmHg, which is associated with diminished background vasopressor requirements and a rapid reduction in renin concentrations when compared to SOC vasopressors. This was particularly notable in patients with RAAS dysfunction in CRS, failing to respond to conventional treatment regimes. Additionally, early initiation after shock recognition may be associated with superior response. Early ATII administration was associated with superior hemodynamic responses at lower doses and in some instances led to a complete cessation for background vasopressors requirements. However, the frequency and sustainability of these findings are poorly reported and has yet to be studied independently. These reporting gaps limited synthesis and contributed to knowledge gaps within the systematic reviews. Patients with pharmacologic suppression of RAAS, typically due to treatment with (angiotensin-converting enzyme inhibitors (ACEi) or angiotensin receptor blockers (ARBs) may require higher doses of ATII to achieve MAP responses. Prior ARB exposure particularly may attenuate responsiveness to ATII and should be considered in clinical decision making.

Despite a higher number of thromboses identified in the ATII arm in the ATHOS-3 RCT, our overview found that the occurrences of adverse events with ATII administration was not higher than in controls. It should be noted that these findings are largely based on post-hoc analysis of ATHOS-3. It is important to recognize that protocolized use of ATII in RCTs was set for 48 h, yet many patients received significantly shorter infusions. In contrast, thromboembolic outcomes were assessed over periods of up to 30 days or until hospital discharge [20]. High-dose catecholamine monotherapy increases the risk of arrythmias, while risk of thrombosis and AKI are documented complications of distributive shock, catecholamine use, and ICU hospitalization [18]. However, this should not preclude caution and use of prophylaxis against venous thromboembolism in the vulnerable population. Interestingly, ATII administration in AKI patients was associated with superior RRT liberation and mortality outcomes. This was further demonstrated in patients with stage 3 AKI [26].

The outcome of interest with the most variability was mortality. Despite 5 of the 6 reviews evaluating mortality, no significant between-group differences were observed. We noted that ATII was primarily used as a third-line agent in patients who remained refractory to high-dose NED > 200 ng/kg/min between 6 and 24 h. However, thresholds for ATII initiation were poorly reported in primary studies and thus reflected in the reviews. It is posited that ATII may have been more liberally prescribed as a rescue intervention in critically ill CRS patients with suspected endogenous decatecholaminization, who were anticipated to have worse prognoses [20, 27]. High-dose catecholamine has been identified as independent predictors of mortality, [18] the catecholamine sparing effects may further benefit survivability in cases of adrenergic receptor saturation from escalating NE and epinephrine administration. Nonetheless, survival rates appear to be higher in specific sub-groups: patients receiving RRT, those with elevated plasma renin concentration, and patients with low NED requirements at the time of ATII initiation exhibited early hemodynamic responses which correlated with improved mortality rates.

This overview underlines the value of biomarkers in ATII guided therapy. Serum biomarkers identifying ACE deficiency from RAAS dysfunction provide a biological rational for individualized care and have been associated with mortality predictability. These biomarkers may also prognosticate as to which patients will benefit from ATII administration early in the disease state. Additional benefits of ATII include improvement in oxygenation, renal perfusion, bacterial clearance, and facilitated lactate reduction while enhancing vascular adrenergic sensitivity [21, 28–31]. Collectively, these findings may indirectly improve long-term survivability outcomes in distributive shock management. We acknowledge that non-adrenergic vasopressors also possess some risks of adverse effects and switching from adrenergic to non-adrenergic vasopressors is not a universal approach. To minimize adverse event occurrence, drug choice, dosing, and timing should be personalized for each patient based on response prognostication.

### Implications for practice

Significant gaps remain in ATII research for distributive shock, particularly regarding timing of initiation, titration strategies, dose response, biomarker guided therapy, and standardized administration, with most studies reproducing the ATHOS-3 trials administration practices. While we recognized that point-of-care (POC) testing for renin or any RAAS biomarkers is currently not commercially available, future research should seek to collaborate with POC manufacturers in development of specific biomarker analysis availability. Evidence is limited on MAP sustainability, hemodynamic relapse, background vasopressor needs, and long-term mortality beyond 28 days. It is recognized that gaps identified within systematic reviews may not fully reflect corresponding knowledge gaps in the included primary studies. Nevertheless, systematic reviews should transparently report such limitations to guide both future research and clinical interpretation. In current practice, the consistent hemodynamic response observed across reviews suggests that ATII may have a role as an adjunct vasopressor in refractory distributive shock; however, variability in definitions of refractory shock and thresholds for initiating non-catecholamine vasopressors continues to limit consistent protocolized use and guideline adoption. High-quality RCT’s are therefore needed to clarify the clinical benefits of ATII, explore biomarker-guided therapy, and evaluate earlier initiation strategies and de-escalation practices. To contextualize these ongoing developments, a list of currently registered protocols for systematic reviews and clinical trials is provided in Appendix G.

### Limitations

The overview is primarily limited by the low quality of included systematic reviews. Quality of evidence could be enhanced if complementary data were available. Given that methodologic deficiencies were generally common to included reviews, we did not adjust or exclude reviews of lower quality or comprised primary studies with higher risk of bias. Rather, we have highlighted these to facilitate appropriate interpretation of findings. To capture relevant reviews, we searched multiple databases following a peer-reviewed protocol and search strategy. Despite this rigorous process, included systematic reviews presented outlying out-of-date primary studies which required weight assessment by the reviewers. After careful assessment, it was determined that these studies did not significantly influence the analysis or overall results of the systematic review, and the weight was deemed minimal. Nonetheless, given their inclusion, the findings from these systematic reviews should be interpreted with caution. Overlap measured by CCA was generally moderate to high for each clinical outcome studied, and primary study overlap amongst reviews was also very high, introducing a problem of precision sampling and reduced reliability. This is attributed to paucity in empirical evidence, with few RCTs and observational studies, primarily derived from small patient populations assessing physiological endpoints. Lastly, most post-hoc analyses within the reviews stem from the ATHOS-3 trial, limiting data diversity. Thus, as is typical of overviews, our findings are more strongly impacted by primary studies found in multiple reviews.

## Conclusion

This overview of reviews on the evidence on angiotensin II use in distributive shock found that in the most severe form of distributive shock, the hemodynamic benefits of ATII administration reduce vasopressor requirements without an associated increase in harm. The overview supports the early addition of ATII in the armamentarium of distributive shock management as it possesses logistical and patient care benefits that are both novel and relevant for future research. However, quantitative studies on the effects of ATII on mortality demonstrated variability in effect size and significance of benefits. This overview also revealed a rather limited number of primary publications, corresponding to considerable overlap in primary studies. This overview supports the need for trials exploring the benefits identified associated with superior mortality benefits.

## Supplementary Information

Supplementary Material 1. [ 4, 6, 7, 11, 12, 15, 16, 21, 23–44].

## Data Availability

Supplemental information can be found in Appendix section. Additional information, including template data collection forms; data collected from included systematic reviews and supplemental primary studies; any other materials used in the overview of reviews can be made available with lead author correspondence.
