# Supplementary material for: An overview of reviews of Angiotensin II in distributive shock
Source: BMC Anesthesiol. 2026 Apr 6;26:257. doi: 10.1186/s12871-026-03797-w (PMC13104260; doi:10.1186/s12871-026-03797-w)
Supplement: Supplementary file 1 — Supplementary Material 1. [ 4, 6, 7, 11, 12, 15, 16, 21, 23–44]. [file 12871_2026_3797_MOESM1_ESM.docx]

Appendix

Appendix A

Reporting guidelines for overview of reviews of healthcare interventions: PRIOR Checklist [6]


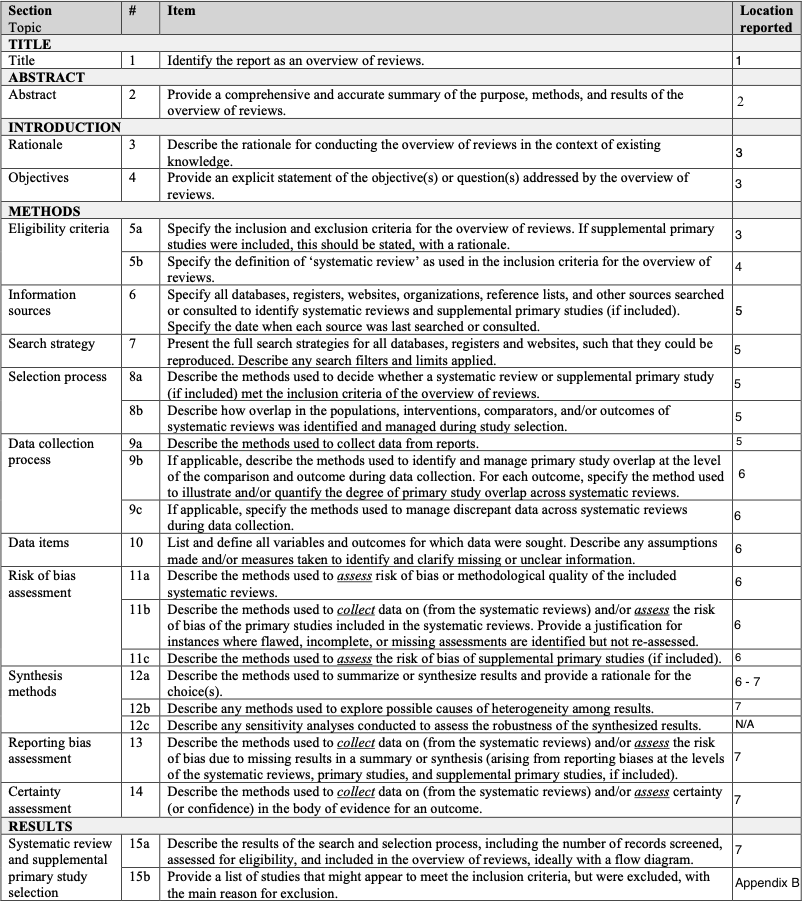


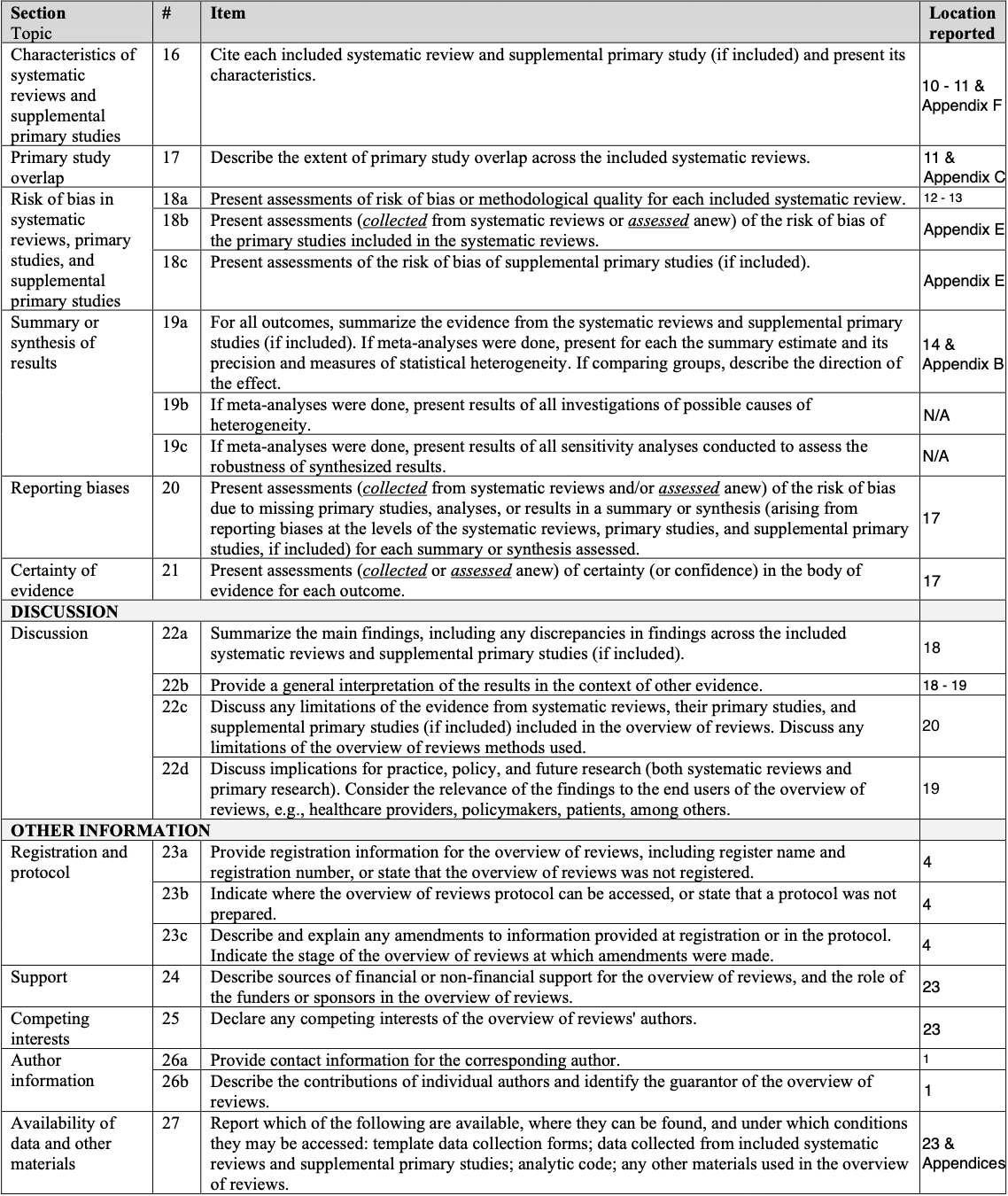


Appendix B

This overview of reviews was based on Preferred Reporting Items for Systematic Reviews and Meta-analyses (PRISMA) Guideline [15]. A comprehensive literature search was performed on seven databases: PubMed, Embase, Cinahl, Epistemonikos, Scopus, PROSPERO, and Cochrane using the following Medical Subject Heading [MeSH] terms in independent and combined searches.

Search 1: (Angiotensin II, angiotensin 2, angiotensin type 2, ATII, AT2, ANG 2)

Search 2: (Distributive shock, Vasodilatory Shock, vasoplegia, Septic Shock, Hypotension, Shock, Cardiopulmonary bypass, post-CPB, post-cardiopulmonary bypass, post-CPB vasoplegia).

Relevant keyword variations and free text word [tw] variations for these concepts were also used. We also isolated searches to systematic reviews both as tw and filter in our search strategy. Below is a combination of MeSH terms and tw used to source out databases. We used Rayyan AI [7] to gather and sort the gathered articles. Rayyan AI was used to ensure publications date guidelines were adhered to, duplicates were automatically detected, manually reviewed, and deleted. Remaining articles we manually screened for eligibility.

Pubmed

Search 1: 86,400
Search 2: 548,905
Combined: 2,564
Systematic Reviews: 32

Embase

Search 1: 97, 611
Search 2: 902,497
Combined: 148

Systematic Reviews: 59

Cinahl

Search 1: 11,471
Search 2: 92,803
Combined: 460
Systematic Reviews: 22

Epistemonikos

Search 1: 8,451
Search 2: 53,612
Combined: 1
Systematic Reviews: 31

Scopus

Search 1: 157,396
Search 2: 759,428
Combined: 3,867

Systematic Reviews: 48

PROSPERO

Search 1: 326
Search 2: 6982
Combined: 36
Systematic Reviews: 36

Cochrane
Search 1: 14,508
Search 2: 23,930
Combined: 10
Systematic Reviews: 4

After date adjustments of 2017 and English language filters applied, 239 articles remained. After duplicates removed 138 articles were initially screened of which 15 were sought out for retrieval. 1 article was not relevant, 1 was an ongoing review, 2 reviews were based on out-of-date studies, and 5 presented wrong intervention or patient population, detailed table below.

Excluded reviews and justifications.

| Author | Review Name | Reason for exclusion |
| --- | --- | --- |
| Khatib et al. 2013 | Blockade of the renin angiotensin system for primary prevention of non-valvular atrial fibrillation: a systematic review and meta-analysis of randomized controlled trials | Wrong intervention and patient population. Out-of-date. |
| Hollmann et al. 2018 | A systematic review of outcomes associated with withholding angiotensin-converting enzyme inhibitors (ACE-I) and angiotensin receptor blockers (ARB) prior to noncardiac surgery | Not relevant. |
| Salahia et al. 2017 | Efficacy and safety of the angiotensin II receptor blocker losartan for hypertrophic cardiomyopathy: Systematic Review and Meta-Analysis | Wrong patient population - Not appropriate patient population for intervention. |
| Prutsky et al. 2018 | Vasopressors in pediatric fluid refractory septic shock: a systematic review and network meta-analysis | Wrong patient population. |
| Um et al. 2019 | Vasopressors in distributive shock: a systematic review and network meta-analysis | Review ongoing. |
| Rodriguez et al. 2020 | Novel Vasopressors in the Treatment of Vasodilatory Shock: A Systematic Review of Angiotensin II, Selepressin, and Terlipressin | Primary studies out-of-date range, unable to isolate as per pre-determined decision rule. |
| Shrestha et al. 2023 | Prior Use of Angiotensin-converting Enzyme Inhibitors or Angiotensin II Receptor Blockers and Clinical Outcomes of Sepsis and Septic Shock: A Systematic Review and Meta-analysis | Not appropriate patient population for intervention. |
| Busse et al. 2017 | Clinical Experience With IV Angiotensin II Administration: A Systematic Review of Safety | Primary studies out-of-date range does, not meet pre-determined decision rule. Intervention not specific to patient population. |
| Bansal et al. 2023 | Efficacy and safety of angiotensin II in cardiogenic shock: A systematic review | Wrong patient population (not distributive shock) |


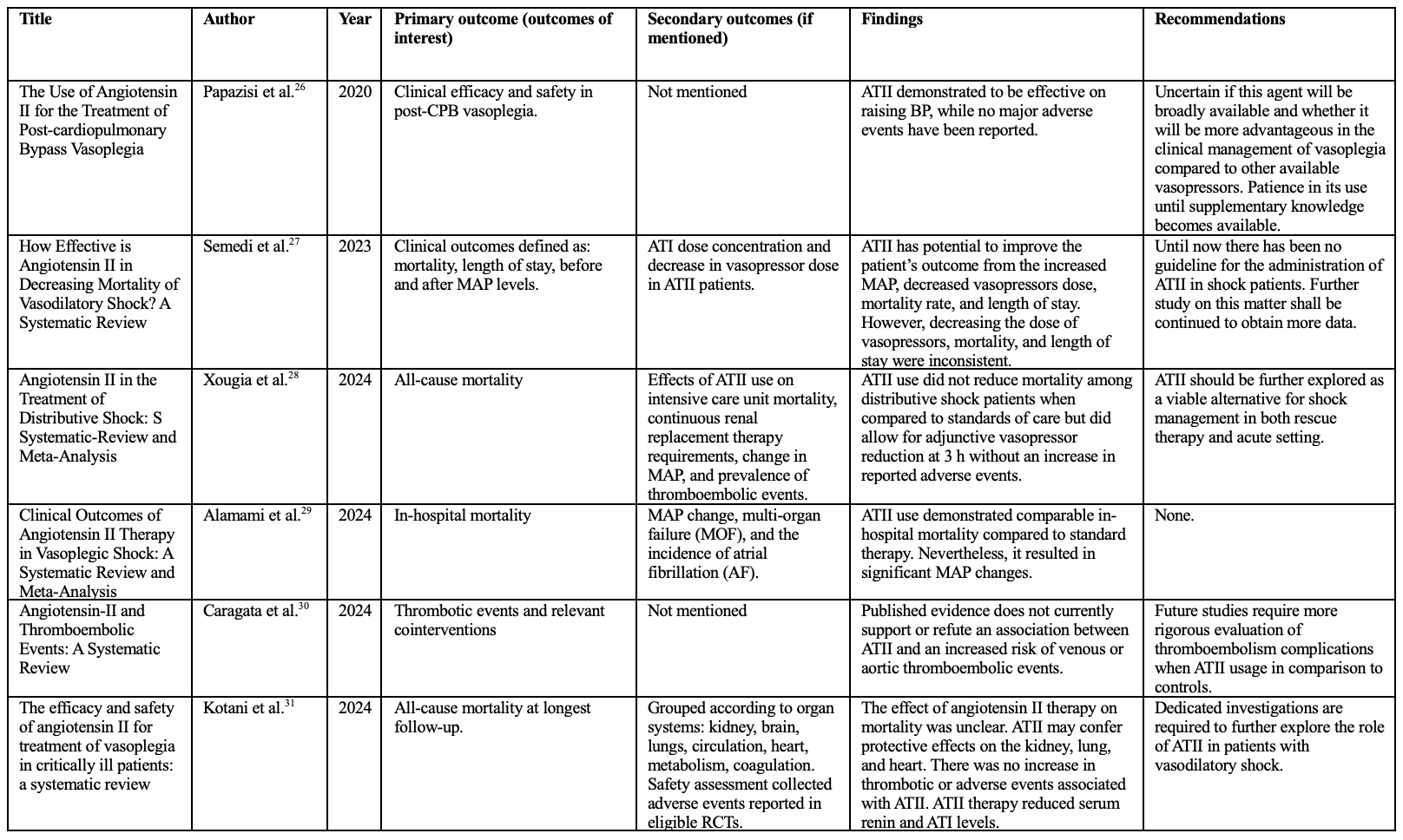


Primary and secondary outcomes of systematic reviews – Findings and recommendations

Appendix C

Systematic review – overlap of primary studies [32–35,4,36,28,37,25,24,38,23,39–43]

| Studies |  |  |  |  |  |  |
| --- | --- | --- | --- | --- | --- | --- |
|  | Papazisi et al. [16] | Semedi et al. [17] | Alamami et al. [19] | Xourgia et al. [18] | Caragata et al.[20] | Kotani et al.[21] |
| Klijian et al. |  |  |  |  |  |  |
| Wieruszewski et al. 2019 |  |  |  |  |  |  |
| Khanna et al. |  |  |  |  |  |  |
| Quan et al. |  |  |  |  |  |  |
| Wieruszewski et al. 2021 |  |  |  |  |  |  |
| Smith et al. |  |  |  |  |  |  |
| See et al. |  |  |  |  |  |  |
| Cutler et al. |  |  |  |  |  |  |
| Bird et al. |  |  |  |  |  |  |
| Wieruszewski et al. 2023 |  |  |  |  |  |  |
| Coulson et al. |  |  |  |  |  |  |
| Zangrillo et al. |  |  |  |  |  |  |
| Chawla et al. |  |  |  |  |  |  |
| Leisman et al. |  |  |  |  |  |  |
| Evans et al. |  |  |  |  |  |  |
| Bennett et al. |  |  |  |  |  |  |
| Geary et al. * |  |  |  |  |  |  |
| Thaker et al. * |  |  |  |  |  |  |
| Sadjadi et al. |  |  |  |  |  |  |
| Meersch et al. |  |  |  |  |  |  |
| Serpa Neto et al. |  |  |  |  |  |  |
| Ten Lohuis et al. |  |  |  |  |  |  |
| Bailey et al. |  |  |  |  |  |  |
| Bobeck et al. |  |  |  |  |  |  |
| Bui et al. |  |  |  |  |  |  |
| Coleman et al. |  |  |  |  |  |  |
| Trethowan et al. |  |  |  |  |  |  |
| Wang et al. |  |  |  |  |  |  |
| Chandra et al. |  |  |  |  |  |  |
| Johnson et al. |  |  |  |  |  |  |
| Šribar et al. |  |  |  |  |  |  |
| Tezel et al. |  |  |  |  |  |  |
| Morselli et al. |  |  |  |  |  |  |
| Blankenship et al. |  |  |  |  |  |  |

**Note:** Included are the primary studies and post-hoc analysis overlap chart.
*Unable to retrieve primary study.

**Corrected covered area (CCA) is calculated following the GROOVE2.0 tool calculator. The equation is calculated as presented below.**


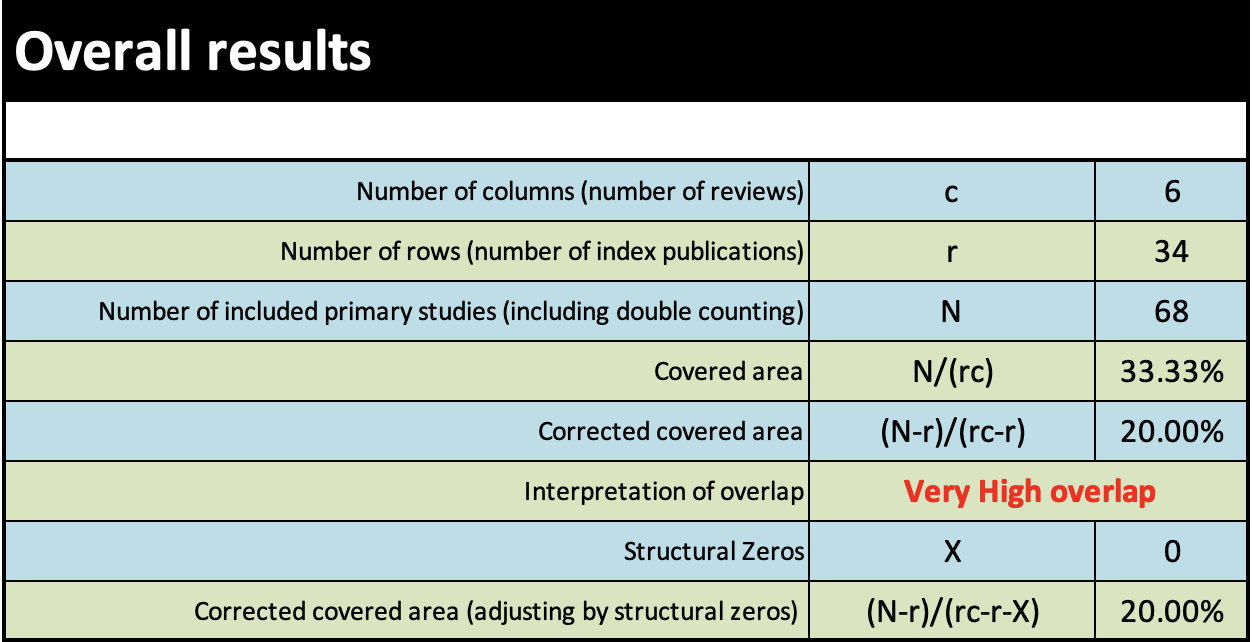


**Systematic review overlap of primary studies**


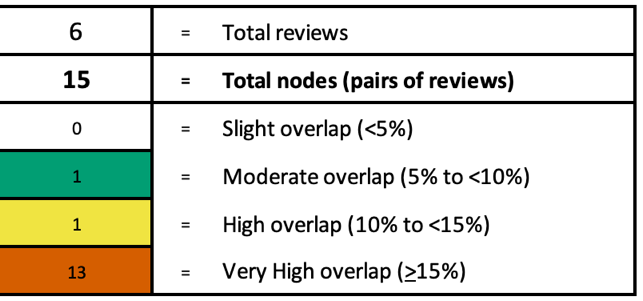


**Extent of primary study overlap within the included systematic reviews**
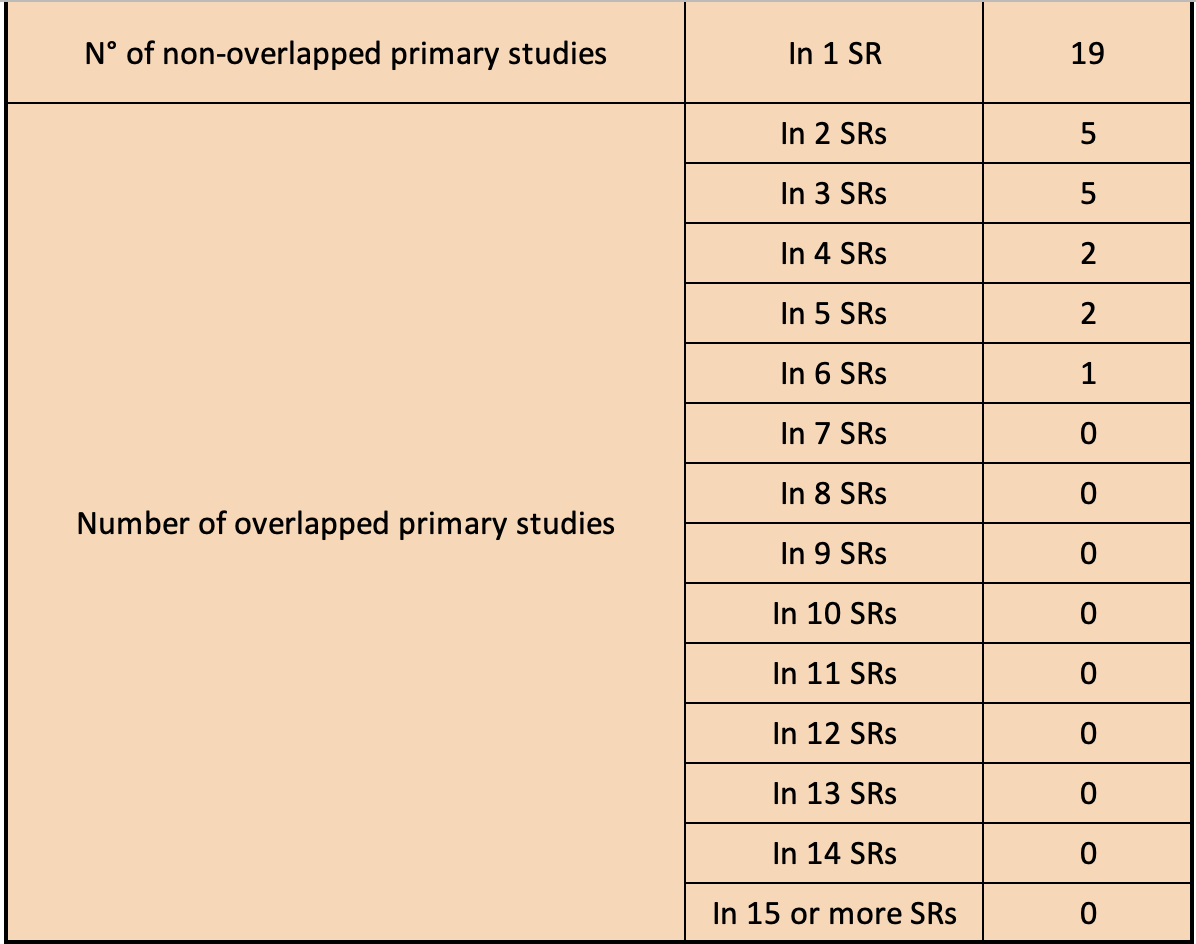


Appendix D

List of AMSTAR2 questions

Q1. Did the research questions and inclusion criteria for the review include the components of PICO?

Q2. Did the report of the review contain an explicit statement that the review methods were established prior to the conduct of the review and did the report justify any significant deviations from the protocol?

Q3. Did the review authors explain their selection of the study designs for inclusion in the review?

Q4. Did the review authors use a comprehensive literature search strategy?

Q5. Did the review authors perform study selection in duplicate?

Q6. Did the review authors perform data extraction in duplicate?

Q7. Did the review authors provide a list of excluded studies and justify the exclusions?

Q8. Did the review authors describe the included studies in adequate detail?

Q9. Did the review authors use a satisfactory technique for assessing the risk of bias (ROB) in individual studies that were included in the review?

Q10. Did the review authors report on the sources of funding for the studies included in the review?

Q11. If meta-analysis was performed did the review authors use appropriate methods for statistical combination of results?

Q12. If meta-analysis was performed, did the review authors assess the potential impact of ROB in individual studies on the results of the meta-analysis or other evidence synthesis?

Q13. Did the review authors account for ROB in individual studies when interpreting/discussing the results of the review?

Q14. Did the review authors provide a satisfactory explanation for, and discussion of, any heterogeneity observed in the results of the review?

Q15. If they performed quantitative synthesis did the review authors carry out an adequate investigation of publication bias (small study bias) and discuss its likely impact on the results of the review?

Q16. Did the review authors report any potential sources of conflict of interest, including any funding they received for conducting the review?

Authors note: Almost all authors defined PICO adequately and explained their rationale behind study selection. However, one review did not mention the comparators in the eligibility criteria. Not every author declared their funding sources and conflicts of interest, none of them reported the funding sources of their primary studies. Most authors comprehensively reported the literature search strategy, searching for grey literature and clinical trials, using experienced consultants in the field. Yet, conducting the search within 24 months of completing the review was a common lack in every review, and none of the author reported the excluded articles with the reason for exclusion. However, most reviews selected and reviewed the studies in duplicate.

Appendix E

Below is the collected risk of bias assessments provided in the systematic reviews. They are presented as found for transparency of reporting framework. The ROB2 tool [12] is a comprehensive framework composed of 5 domains. (1) Bias arising from randomization process, (2) Bias due to deviations from intended intervention, (3) Bias due to missing outcome data, (4) Bias in the measurement of the outcome, (5) Bias in the selection of the reported result. Each domain is concluded with concern rating scale of low, some concerns, and high. For observational studies, the ROBINS-I tool [11] was used. This tool is comprised of seven domains, the overall risk assigned as low if judged as low provided this was assigned in all domains, moderate if the risk of bias was assessed as low or moderate in all domains, serious if the risk of bias was serious in at least one of the domains, critical if the risk of bias was critical in a minimum of one domain, and no information in the absence of information in one or more domains.

| Author | D1 | D2 | D3 | D4 | D5 | Overall |
| --- | --- | --- | --- | --- | --- | --- |
| Khanna et al. [4] 2017 |  |  |  |  |  |  |
| Chawla et al. [35] 2014 |  |  |  |  |  |  |
| Bennet et al. 2001 [43] |  |  |  |  |  |  |
| Coulson et al. [38] 2022 |  |  |  |  |  |  |

ROB2 reported by systematic review authors

*Unable to retrieve primary study.

**Abbreviations:**

D1 – Randomisation process

D2 – Deviation from the intended interventions

D3 – Missing outcome data

D4 – Measurement of the outcome

D5 – Selection of the reported result

Judgement

Low risk of bias

Potential risk of bias

High risk of bias

| Author | Confounding | Selection of Participants | Classification of Intervention | Deviation from Intended Interventions | Missing Data | Measurement of Outcomes | Selection of Reported Results | Overall |
| --- | --- | --- | --- | --- | --- | --- | --- | --- |
| Wieruszewski et al. [25] 2021 |  |  |  |  |  |  |  |  |
| Wieruszewski et al. [36] 2023 |  |  |  |  |  |  |  |  |
| Smith et al. [23] 2022 |  |  |  |  |  |  |  |  |
| See et al. [39] 2023 |  |  |  |  |  |  |  |  |
| Zangrillo et al. [37] 2020 |  |  |  |  |  |  |  |  |
| Klijian et al. [42] 2021 |  |  |  |  |  |  |  |  |

ROBINS-I reported by systematic review authors.

Judgement

Critical

Serious

Low

No Information

Moderate

The below is provided by the reviewers (MD and HA) for conflicting or absent results in risk of bias of the included studies in the systematic reviews. Being unable to obtain the assessment tool from the authors, or when discrepancies were found in results, a decision was made to perform ROB anew. The ROBINS-I assessment tool [11] was used for observational studies.

| Author | Confounding | Selection of Participants | Classification of Intervention | Deviation from Intended Interventions | Missing Data | Measurement of Outcomes | Selection of Reported Results | Overall |
| --- | --- | --- | --- | --- | --- | --- | --- | --- |
| Bird et al. [33] 2022 |  |  |  |  |  |  |  |  |
| Cutler et al. [44] 2020 |  |  |  |  |  |  |  |  |
| Evans et al. [41] 2019 |  |  |  |  |  |  |  |  |
| Quan et al. [24]2022 |  |  |  |  |  |  |  |  |
| Leisman et al. [40] 2020 |  |  |  |  |  |  |  |  |
| Wieruszewski et al. [28] 2019 |  |  |  |  |  |  |  |  |
| Geary et al. 1990* |  |  |  |  |  |  |  |  |
| Thaker et al. 1990* |  |  |  |  |  |  |  |  |
| Serpa Neto et al. 2022 |  |  |  |  |  |  |  |  |
| Meersch et al. 2022 |  |  |  |  |  |  |  |  |
| Blanckenship et al. 2024 |  |  |  |  |  |  |  |  |

*Cochrane ROBINS-I quality assessment tool*

**Study not retrieved due to lack of access – ROB not reported by author.*

Judgement

Critical

Serious

Moderate

Low

No Information

Appendix F

Primary studies characteristics [4,32–36,28,37,25,24,38,23,39–43]

| **Author** | **Year** | **Design** | **Patient Population** | **Cause of Distributive Shock receiving ATII** |
| --- | --- | --- | --- | --- |
| Zangrillo et al. | 2020 | Case Series | 15 | Septic Shock n=15 |
| Wieruszewski et al. | 2021 | Multicenter Retrospective Cohort | 270 | Septic Shock n=149 Post-CPB Vasoplegia n=28 Other n=46 |
| Khanna et al. | 2017 | Multicenter Clinical Trial - RCT | 344 | Septic Shock n=147 Post-CPB Vasoplegia n=10 Other n=6 |
| Quan et al. | 2022 | Multicenter Retrospective Cohort | 147 | Septic Shock n=51 |
| Coulson et al. | 2022 | Multicenter Double-blind Randomised Feasibility Trial | 60 | Post-CPB Vasoplegia n=28 |
| Smith et al. | 2022 | Multicenter Retrospective Cohort | 162 | Septic Shock n=117 Other n=45 |
| See et al. | 2023 | Single Center Clinical Trial – Non-Randomized | 120 | Septic Shock n=24 Post-CPB Vasoplegia n=6 Other n=10 |
| Cutler et al. | 2021 | Case Report | 5 | Post-CPB Vasoplegia n=5 |
| Bird et al. | 2023 | Retrospective Review | 19 | Post-CPB Vasoplegia n=7 |
| Wieruszewski et al. | 2019 | Case Report | 4 | Post-CPB Vasoplegia n=4 |
| Chawla et al. | 2014 | Single Center Clinical Trial -RCT | 20 | Distributive Shock (all cause) n=20 |
| Leisman et al. | 2020 | Multicenter Retrospective Observational | 29 | Distributive Shock (Covid-19) n=29 |
| Evans et al. | 2019 | Case Report | 1 | Post-CPB Vasoplegia n=1 |
| Bennet et al.* | 2001 | Single Center Clinical Trial RCT | 10 | Post CPB n=10 |
| Geary et al.* | 1990 | Case Report | 1 | N/A |
| Thake et al. * | 1990 | Case Report | 1 | N/A |
| Sadjadi et al. | 2024 | Single Center Clinical Trial RCT | 63 | Post-CPB n=32 |
| Meersch et al. | 2022 | Retrospective Single Center Trial | 40 | Post-CPB n=20 |
| Serpa Neto et al. | 2022 | Multicenter Prospective Trial | 132 | Covid-19 n=65 |
| Ten Lohuis et al. | 2022 | Retrospective Study | 78 | Post-CPB n=78 |
| Bailey et al. | 2019 | Case Report | 2 | Septic Shock n=2 |
| Bobeck et al. | 2020 | Case Report | 1 | Covid-19 n=1 |
| Bui et al. | 2020 | Case Report | 1 | Septic Shock n=1 |
| Coleman et al. | 2020 | Case Report | 1 | Septic Shock n=1 |
| Trethowan et al. | 2020 | Case Report | 1 | Post-CPB n=1 |
| Wang et al. | 2020 | Case Report | 1 | Covid-19 n=1 |
| Chandra et al. | 2021 | Case Report | 1 | Septic Shock n=1 |
| Johnson et al. | 2023 | Single Center Retrospective Observational Study | 30 | Post-CPB n=30 |
| Šribar et al. | 2023 | Case Report | 1 | Post-CPB n=1 |
| Tezel et al. | 2023 | Single Center Retrospective Case series | 23 | Distributive Shock n=23 |
| Morselli et al. | 2020 | Case Series | 7 | Covid-19 n=7 |
| Blankenship et al. | 2024 | Propensity-Matched Cohort Study | 69 | Septic Shock n=23 |

**Notes:** Patient populations are encompassing both control and intervention groups. Causes of distributive shock receiving ATII section is solely representative of the intervention group withing the patient population.

**Abbreviations:** CPB = Cardio-Pulmonary Bypass surgery, RCT = Randomized Control Trial, N/A = Not Available.

*Unable to retrieve / outdated publication – information retrieved from systematic review.

Characteristics of Post-Hoc Analysis’.

| **Author** | **Year** | **Study Reviewed** | **Review Details** |
| --- | --- | --- | --- |
| Wieruszewski et al. [36] | 2023 | ATHOS-3 | ATII initiation at lower vasopressor doses |
| Klijian et al. [42] | 2021 | ATHOS-3 | ATII in CPB and vasopressor sparing capabilities |

**Abbreviations:** CPB = Cardio-Pulmonary Bypass surgery.

Appendix G

The provided is the current results and status of registered protocols for systematic reviews, meta-analysis, and clinical trials exploring Angiotensin II use is shock states.

PROSPERO
Vasopressors in distributive shock: a systematic review and network meta-analysis: CRD42019139849 – Review Ongoing.

Angiotensin II in vasodilatory shock: meta-analysis of randomised controlled trials: CRD42022364848 – Review Ongoing.

Angiotensin II in the management of distributive shock: CRD42022369289 – Review Ongoing.

Analysis of Clinical Experience with Angiotensin II: A Meta-Analysis and Four IAs: CRD42024578717 – Review Ongoing. (Spanish only).

ClinicalTrials.gov

Serum Biomarkers to Predict Response to Angiotensin II in Septic Shock: NCT05824767 – Currently recruiting.

Early Angiotensin II in the Emergency Department (ANGIO-ED): NCT06693726 – Not yet recruiting.

Angiotensin II for Distributive Shock: NCT04904562 – Currently recruiting.

Hemodynamic Response to Angiotensin-II When Used as the Second Vasopressor Agent for Septic Shock: NCT06122987 – Currently recruiting.

Efficacy and Safety of Angiotensin II Injection Versus Placebo in Patients With Refractory Distributed Shock: NCT06351150 – Currently recruiting.

ANZCTR

Acute Renal effects of Angiotensin II Management in Shock (ARAMIS-2): ACTRN12621001043820 – Currently recruiting.
